# The Hypocholesterolemic Effect of Germinated Brown Rice Involves the Upregulation of the Apolipoprotein A1 and Low-Density Lipoprotein Receptor Genes

**DOI:** 10.1155/2013/134694

**Published:** 2013-02-25

**Authors:** Mustapha Umar Imam, Maznah Ismail, Abdul Rahman Omar, Hairuszah Ithnin

**Affiliations:** ^1^Laboratory of Molecular Biomedicine, Institute of Bioscience, Universiti Putra Malaysia, 43400 Serdang, Selangor, Malaysia; ^2^Department of Nutrition and Dietetics, Faculty of Medicine and Health Sciences, Universiti Putra Malaysia, 43400 Serdang, Selangor, Malaysia; ^3^Department of Veterinary Pathology and Microbiology, Faculty of Veterinary Medicine, Universiti Putra Malaysia, 43400 Serdang, Selangor, Malaysia; ^4^Department of Pathology, Faculty of Medicine and Health Sciences, Universiti Putra Malaysia, 43400 Serdang, Selangor, Malaysia

## Abstract

Germinated brown rice (GBR) is rich in bioactive compounds, which confer GBR with many functional properties. Evidence of its hypocholesterolemic effects is emerging, but the exact mechanisms of action and bioactive compounds involved have not been fully documented. Using type 2 diabetic rats, we studied the effects of white rice, GBR, and brown rice (BR) on lipid profile and on the regulation of selected genes involved in cholesterol metabolism. Our results showed that the upregulation of apolipoprotein A1 and low-density lipoprotein receptor genes was involved in the hypocholesterolemic effects of GBR. Additionally, in vitro studies using HEPG2 cells showed that acylated steryl glycoside, gamma amino butyric acid, and oryzanol and phenolic extracts of GBR contribute to the nutrigenomic regulation of these genes. Transcriptional and nontranscriptional mechanisms are likely involved in the overall hypocholesterolemic effects of GBR suggesting that it may have an impact on the prevention and/or management of hypercholesterolemia due to a wide variety of metabolic perturbations. However, there is need to conduct long-term clinical trials to determine the clinical relevance of the hypocholesterolemic effects of GBR determined through animal studies.

## 1. Introduction

Glucose and cholesterol metabolism are closely related, and in individuals with type 2 diabetes, problems related to insulin deficiency/insensitivity result in dyslipidemia, which eventually increases the risk of severe complications [1]. Insulin deficiency and insensitivity result in the dysregulation of cholesterol metabolism, leading to dyslipidemia, (high concentrations of low-density lipoprotein (LDL) cholesterol, and triglycerides and low concentrations of high-density lipoprotein (HDL) cholesterol). Generally, dyslipidemia causes atherosclerosis, which is responsible for cardiovascular disease [2]. Cardiovascular disease is the major cause of disability and death in type 2 diabetics. Although atherosclerosis serves as a direct link between dyslipidemia and cardiovascular disease; disturbances in the tightly regulated process of cholesterol metabolism contribute immensely to cardiovascular disease [3]. Several drugs are used to treat dyslipidemia, but, because of their side effects and ultimate failure to satisfactorily manage dyslipidemia, there is an increasing need for alternatives, which are gradually being developed [4, 5]. 

Statins are central to the management of hypercholesterolemia but may slightly increase the risk of type 2 diabetes [6], necessitating the search for more options. The dietary management of dyslipidemia has been shown to be effective, and numerous dietary approaches are becoming popular in the management of hypercholesterolemia [7]. One advantage of dietary management is the lack of drug-induced side effects. White rice (WR) is a staple food for the majority of people in Asia and Africa. Its high glycemic index could promote oxidative stress and cardiovascular complications, especially in individuals with type 2 diabetics [8, 9], making its consumption unsuitable. Germinated brown rice (GBR) has been reported to lower cholesterol [10–14], and the effects of its individual bioactive components may contribute to its overall effects on cholesterol. Phytosterol glycosides and oryzanol are known to lower cholesterol [15, 16], and gamma amino butyric acid (GABA) was suggested by Roohinejad et al. to be the primary component in GBR responsible for hypocholesterolemia [13]. Other bioactive compounds in GBR like oryzanol have been reported to have hypocholesterolemic effects [17], and it is likely that the hypocholesterolemic effects of GBR result from cumulative effects of these bioactive compounds. Several mechanisms are apparently involved in the hypocholesterolemic effects of GBR. We studied the effects of GBR and select bioactive compounds on hypercholesterolemia and on the regulation of cholesterol metabolism, which was hypothesized to be a likely mechanism for the hypocholesterolemic effect of GBR. The most important aspects of cholesterol metabolism are regulated at the transcriptional level, and the dysregulation of metabolism in type 2 diabetics affects many molecular pathways, including those related to cholesterol metabolism. Thus we studied the effects of GBR on the expression of apolipoprotein A1 (APO A1), low-density lipoprotein receptor (LDL-R), and 3-hydroxy-3-methylglutaryl coenzyme A reductase (HMGCR).

## 2. Materials and Methods

### 2.1. Materials

All solvents were of analytical grade and were purchased from Merck (Darmstadt, Germany). GABA, oryzanol, phenolics standards, streptozotocin (STZ), Tris-EDTA (TE) buffer solution, dexamethasone, N,O-bis(trimethylsilyl)trifluoroacetamide with 1% trimethylchlorosilane (BSTFA + 1% TMCS), RPMI 1640 medium, fetal bovine serum, and antibiotics were purchased from Sigma-Aldrich. The ASG standard was purchased from Matreya (USA), and the glucose assay and lipid profile kits were obtained from Randox Laboratories Ltd. (Crumlin, County Antrim, UK). Insulin was purchased from Invitrogen, and other cell culture materials were purchased from BD Biosciences (NJ, USA). The GenomeLab GeXP Start Kit was obtained from Beckman Coulter Inc. (USA), and the RNA isolation kit (GF-TR-100 RNA Isolation Kit) was supplied by Vivantis (Selangor, Malaysia). RCL 2 was purchased from Alphelys (Toulouse, France), and MgCl_2_ and DNA Taq polymerase were purchased from Thermo Fisher Scientific (Pittsburgh, PA). Hydrogen peroxide (H_2_O_2_) was obtained from Bendosen Laboratory Chemicals (Selangor, Malaysia), and sodium hypochlorite was purchased from Dexchem Industries Sdn. Bhd., (Penang, Malaysia). The fine sugar and starch powders used to make pellets were purchased from R & S Marketing Sdn. Bhd. (Malaysia), and the Mazola oil, Nespray fortified milk powder, and standard rat chow were obtained from Unilever (Malaysia), Nestle Manufacturing (Malaysia), and Specialty Feeds (TN, USA), respectively.

### 2.2. Germination of Brown Rice and Preparation of Bioactive Compounds

The germination of brown rice (BR) and the preparation of total phenolic extracts were reported in our earlier publication [18]. Acylated steryl glycoside (ASG) was extracted as reported by Usuki et al. [19] and analyzed by GC-MS/MS QQQ (Thermo Fischer Scientific, Logan, CA) using the method described by Phillips et al. [20]. GABA was extracted and analyzed by HPLC-DAD as reported by Rozan et al. [21]. Oryzanol was prepared as reported by Azrina et al. [22]. Pellets were made from WR, BR, and GBR for animal feeding.

### 2.3. Animal Study

Sprague-Dawley rats (30, male) between 150 and 200 g each were housed in individual plastic cages under controlled conditions (25–30°C with a 12/12 h light/dark cycle). Guidelines for the use of animals were strictly adhered to and were approved by the Animal Care and Use Committee (ACUC) of the Faculty of Medicine and Health Sciences, Universiti Putra Malaysia (project approval number UPM/FPSK/PADS/BR-UUH/00360). After a 2-week period of adaptation with standard rat chow available ad libitum and free access to water, the normal nondiabetic rats (5) were maintained on standard rat chow. The rest of the rats was fed a high fat diet (HFD) for 6 weeks to induce obesity and then injected with streptozotocin (STZ) (35 mg/kg b.w.; i.p) to induce type 2 diabetes, as reported in our earlier publication. Diabetic rats (fasting blood glucose of ≥250 mg/dL after 2 days of STZ) were randomly divided into 5 groups (5 rats each); the control (diabetic untreated) group received the HFD, the WR group received an HFD in which 50% of the normal rat chow was substituted with WR, the BR group received an HFD in which 50% of the normal rat chow was substituted with BR, the G50 group received an HFD in which 50% of the normal rat chow was substituted with GBR, and the G100 group received a HFD in which 100% of the normal rat chow was substituted with GBR. The rats were maintained on these diets for 28 days. Food consumption was measured daily by weighing the leftover food and subtracting this weight from the total pellet weight given the previous day.

### 2.4. Glucose and Lipid Profile Analyses

Biochemical analyses were performed in blood collected weekly by venous puncture after an overnight fast. Samples were analyzed using Randox analytical kits according to manufacturer's instructions using a Selectra XL instrument (Vita Scientific, Dieren, The Netherlands).

### 2.5. In Vitro Studies

HEPG2 cells from the American Type Culture Collection (Manassas, VA) were cultured in RPMI 1640 medium supplemented with 10% fetal bovine serum (FBS) and 1% antibiotics (100 U/mL penicillin) in an incubator at 37°C with 5% CO_2_. Cell viability was assessed by seeding HEPG2 cells in a 96-well plate at a density of 5 × 10^5^ cells/well and culturing the cells for 24 h at 37°C with 5% CO_2_. Different concentrations (50 ppm–1000 ppm) of extracts (ASG, GABA, oryzanol, and phenolics) were applied for another 24 h, and the cells were then exposed to MTT. The resulting chromogen (formazan) was solubilized using DMSO, and the absorbance was read at 570 nm in a microplate reader. The cell viability was expressed as the percentage of live cells relative to the number of control cells. Cells subcultured on a 24-well plate were allowed to attach for 24 h, after which they were subjected to serum starvation in RPMI 1640 medium supplemented with 0.5% FBS and 1% antibiotics for another 12 h. These cells were treated with nontoxic doses (50 ppm) of GABA (IC50-785 ppm), ASG (IC50-581 ppm), oryzanol (IC50-1066 ppm), total phenolic extracts (IC50-977 ppm), or insulin (100 nM) in a medium that contained 1 *μ*M dexamethasone in addition to 10% FBS and 1% antibiotic. After 24 h of treatment, the medium was removed, and the cells were washed with PBS. The cells were then used for RNA extraction. 

### 2.6. Gene Expression

Primer sequences for all of the rat and human genes except the internal control (KanR) and the housekeeping genes were designed on the NCBI website. The primers for the KanR and housekeeping genes were supplied by Beckman Coulter (USA). The primers listed in Table 1 were supplied by First BASE (Selangor, Malaysia) and reconstituted in 1X TE buffer according to the protocol of the GenomeLab GeXP kit (Beckman Coulter, USA). RNA was extracted from rat livers and HEPG2 cells at the end of the animal and cell culture studies using the GF-TR-100 RNA isolation kit (Vivantis, Selangor, Malaysia) according to the manufacturer's instructions. Reverse transcription and PCR were performed according to the GenomeLab GeXP kit protocol (Beckman Coulter, USA) in an XP Thermal Cycler (Bioer Technology, Germany). The PCR products were finally analyzed with a GeXP genetic analysis system, and the results were normalized using eXpress Profiler software based on the manufacturer's instructions.

### 2.7. Statistical Analysis

The means of the groups were used for the analyses; where error bars are shown, they represent the SDs. One-way analysis of variance (ANOVA) performed using SPSS 17.0 software (SPSS Inc., Chicago, IL, USA) was used to assess the level of significance of differences between means with a cutoff of *P* < 0.05.

## 3. Results

### 3.1. Germinated Brown Rice Bioactive Compounds

The potentiation of bioactive compounds through germination is one way to enhance the benefits of GBR. In the current study, our GBR variety was found to be rich in bioactive compounds. ASG was found to be present at a level of 0.465 ± 0.055 mg/g of GBR, and the GABA concentration was 0.36 ± 0.04 mg/g of GBR. Four isomers of oryzanol (cycloartenyl ferulate, 24-methylene cycloartenyl ferulate, campestryl ferulate, and mixtures of *β*-sitosteryl ferulate and cycloartenyl ferulate) combined were present at 30.38–64.22 mg/100 g of GBR. Phenolics were also found to be present at high levels, and the GBR had a high antioxidant potential, as reported in our earlier publication [18]. The concentrations of ASG, GABA, oryzanol, and phenolics in GBR in comparison to WR and BR are shown on Table 2.

### 3.2. Food Consumption, Plasma Glucose, and Lipid Profile

Table 3 shows the baseline parameters (plasma glucose and lipid profile) and food consumption for each group. Food consumption was similar among all groups. Over the 28 days of the intervention, the fasting plasma glucose level for the normal nondiabetic group was within normal limits, although it increased insignificantly, by approximately 5%. In contrast, the diabetic control group exhibited progressively increasing glucose levels, which were elevated by 19% by the end of the study. The WR group had the highest level of glycemia (28% increase after 28 days), likely due to the high glycemic index of WR. The glycemia levels of the BR, G50 and G100 groups were reduced by 8%, 16%, and 34%, respectively, over the intervention period. 

**Figure 1:**
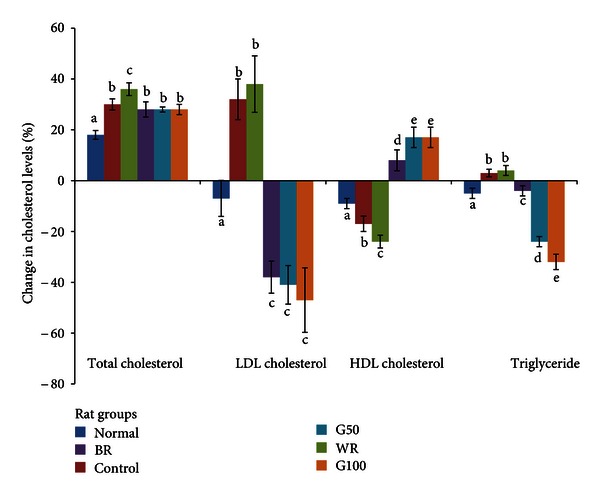
Changes in lipid profile over the 4 weeks of the dietary intervention. The figure shows the effects of germinated brown rice (GBR) on total cholesterol, LDL cholesterol, HDL cholesterol, and triglycerides in type 2 diabetic rats over the 4 weeks of the dietary intervention compared with the effects of brown rice (BR) and white rice (WR) (*n* = 5/group). The data represent the means of each group, and the error bars represent SDs. Bars with the same letters are not significantly different (*P* > 0.05). The control diabetic untreated group received a high fat diet (HFD), and the normal nondiabetic group received standard rat chow. The WR, BR, and G50 groups received an HFD in which 50% of the standard rat chow was substituted with rice, and, in the G100 group, 100% of the rat chow was substituted with GBR.

The changes in lipid profile after 28 days of intervention are shown in Figure 1. At the end of the animal study, the total cholesterol level was significantly higher in the WR group than in the other groups. The increases in the total cholesterol levels in the BR and GBR groups were not significantly different and not as great as those in the WR group. The LDL cholesterol and triglyceride levels were similarly elevated in control and WR groups, whereas the HDL cholesterol levels were reduced in the groups compared with the BR and GBR groups. BR and GBR reduced the levels of LDL cholesterol and triglycerides and improved the HDL cholesterol levels. 

### 3.3. The Effects of GBR and Bioactive Compounds on the Expression of the LDL-R and APO A1 Genes in the Rat Liver and HEPG2 Cells

**Figure 2:**
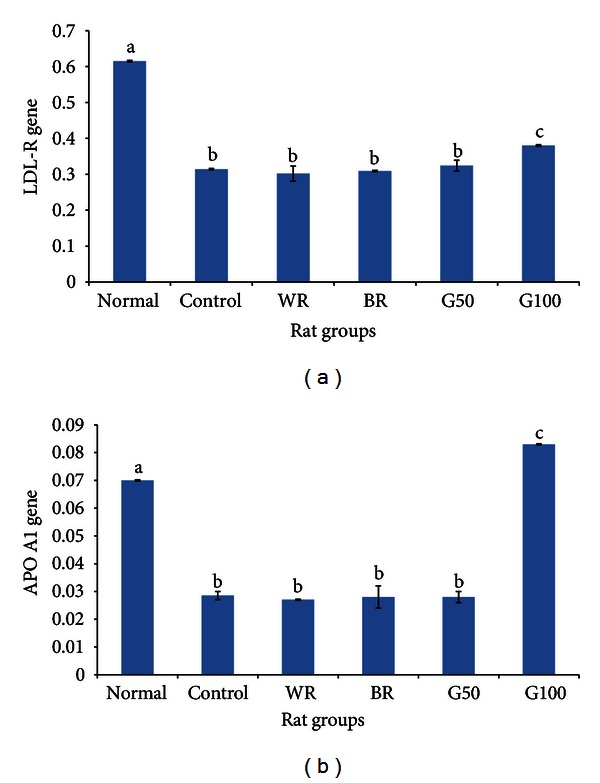
Changes in rat hepatic expression of (a) the low-density lipoprotein receptor (LDL-R) gene and (b) the apolipoprotein A1 (APO A1) gene after 4 weeks of the dietary intervention. The figure shows the effect of germinated brown rice (GBR) on the expression levels of the LDL-R and APO A1 genes in type 2 diabetic rats after 4 weeks of the intervention compared with the effects of brown rice (BR) and white rice (WR) (*n* = 5/group). The data represent the means of each group, and the error bars represent the SDs. Bars with different letters are significantly different (*P* < 0.05). The groupings are the same as in Figure 1.

Figures 2(a) and 2(b) show the expression levels of the LDL-R and APO A1 genes in type 2 diabetic rat livers after 28 days of dietary intervention. The level of transcription of the LDL-R gene in the control group was significantly lower than the level in the normal nondiabetic group. The LDL-R expression levels in the WR, BR, and G50 groups were not significantly different from those in the control group, but the LDL-R expression level in the G100 group was higher than the levels in all other groups except the normal nondiabetic group. Similarly, the expression levels of the APO A1 gene were similar among the control, WR, BR, and G50 groups and were significantly lower than the expression level in the normal nondiabetic group. In the case of the APO A1 gene, however, the level of expression in the G100 group was significantly higher than that in all other groups, including the normal nondiabetic group. 

**Figure 3:**
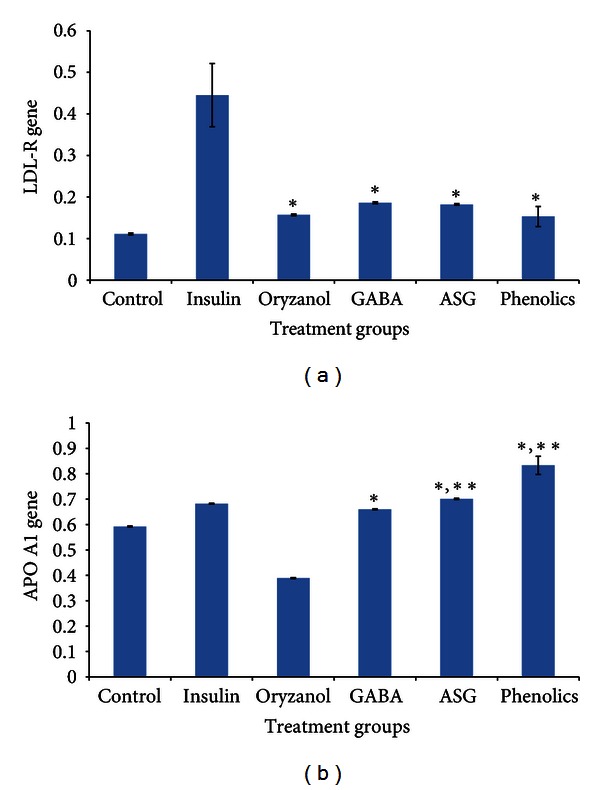
Changes in the expression levels of (a) the low-density lipoprotein receptor (LDL-R) gene and (b) the apolipoprotein A1 (APO A1) genes in HEPG2 cells following 24 h of treatment. The figure shows the effect of 50 ppm of acylated steryl glycoside (ASG), *γ*-amino butyric acid (GABA), oryzanol, and phenolic extracts on the expression levels of LDL-R and APO A1 genes in HEPG2 cells following 24 h of treatment compared with the effects of insulin (100 nM) and no treatment (*n* = 4). The data represent the means of each group, and the error bars represent SDs. *Bioactive group significantly higher than the control group. **Bioactive group significantly higher than the insulin group (*P* < 0.05).

Figures 3(a) and 3(b) show the effects of ASG, GABA, oryzanol, and phenolic extracts from GBR on the expression levels of the LDL-R and APO A1 genes in HEPG2 cells. Following 24 hr of treatment, all extracts upregulated the LDL-R gene significantly with respect to the control level but not as much as insulin, which was presumed to reflect normal physiologic conditions. In the case of the APO A1 gene, however, only ASG, GABA and phenolic extracts significantly upregulated the gene relative to the level in the control group. Treatment with ASG and phenolic extracts resulted in higher expression levels than did insulin treatment. 

## 4. Discussion

### 4.1. GBR and Its Bioactive Compounds

The concentration of bioactive compounds has been shown to increase during the germination of BR [17], and the findings of the current study support these results. Although GBR has been widely reported to have cholesterol-lowering effects, the identities of the bioactive compounds and the mechanisms involved are still the subject of debate. It is likely that the synergistic effects of multiple bioactive compounds produce the overall hypocholesterolemic effects of GBR. Oryzanol has been reported to have strong hypocholesterolemic effects [16]. High amounts of oryzanol would therefore confer a greater functional effect, and germination may be one way of enhancing the concentration of this compound. We found high amounts of oryzanol in our study, suggesting a high functional potential for GBR. The concentration that we found in this study was higher than previously reported [23]. GABA also has hypocholesterolemic effects. Roohinejad et al. suggested that GABA has hypocholesterolemic effects [13]. They reported that high amounts of GABA in GBR increased the hypocholesterolemic effects, and because our GBR contained higher amounts of GABA, it could be argued that our GBR will be more effective in lowering cholesterol. Our GABA concentration was higher than the concentrations reported by others for the same type of rice germinated for the same duration [24]. However, Esa et al. reported that higher amounts of oryzanol, tocopherols, and monounsaturated fatty acids confer better hypocholesterolemic effects [12]. Phytosterol glycosides such as ASG have been shown to have hypocholesterolemic effects, and the mechanism is believed to involve the regulation of cholesterol absorption [15], which eventually lowers cardiovascular disease risk [25]. Additionally, phenolic compounds are reported to have hypocholesterolemic effects in addition to their antioxidant effects [26]. Other bioactive compounds, such as dietary fiber, may also play a role [27]. Although hypocholesterolemic effects have been shown to correlate positively with some bioactive compounds, little is known about how GBR regulates cholesterol metabolism at the transcriptional level. In this study, we focused on the transcriptional regulation of cholesterol metabolism as a potential mechanism for hypocholesterolemia to determine how GBR regulates cholesterol levels and which bioactive compounds contribute to this effect. 

### 4.2. The Hypocholesterolemic Effects of GBR

Cardiovascular diseases are responsible for a great majority of the disability and death among individuals with type 2 diabetes mellitus, mostly due to dyslipidemia [1], and treatment strategies targeted at lowering cholesterol are almost always accompanied by glycemic management to concurrently reduce the risk of cardiovascular complications [28]. Growing evidence for the hypocholesterolemic effects of GBR suggests that when it is consumed, it can prevent or at least delay complications secondary to dyslipidemia, which account for many cases of disability and death in type 2 diabetics. In diabetes, hypertriglyceridemia has been implicated in cardiovascular disease due to type 2 diabetes [1], whereas high levels of HDL cholesterol, the major component of which is APO A1, are believed to be the most important indicator for cardioprotection among the lipid components [29, 30]. Additionally, improvements in HDL cholesterol have been shown to independently reduce the risk of cardiovascular disease [31], and as our data shows GBR has the potential to reduce cholesterol levels in addition to facilitating glycemic control. The pattern of change in the lipid profile reflected in our data suggests that although BR and especially GBR have the same effects on total cholesterol as the control treatment, their effects on triglycerides and HDL cholesterol indicate that they may protect against the adverse effects of dyslipidemia. Although the total cholesterol levels for BR and GBR groups were similar to those in the control group, the levels of individual lipid components were altered in a cardioprotective fashion. And as suggested by the changes in the different components of the lipid profile, the contributions of LDL cholesterol, HDL cholesterol, and triglycerides to the total cholesterol are different in the control, BR and GBR groups. Additionally, the reduction in LDL cholesterol, HDL cholesterol, and triglyceride may have been a result of an innate ability of the rats to regulate their lipid profile or may in effect reflect the natural history of lipid profile changes in normal nondiabetic rats that consume standard rat pellet. Our findings therefore support earlier reports that BR and GBR lower glycemia and improve the lipid profile, and therefore BR and GBR may offer protection against cardiovascular diseases in diabetics.

### 4.3. Effects of GBR on LDL-R and APO A1 Genes

The fact that the expression of the LDL-R gene in the control group was significantly lower than that in the normal nondiabetic group suggests that the protein encoded by this gene may be present at higher concentrations in the normal nondiabetic group than in the control group. This difference could likely explain the higher levels of LDL in the control group because LDL-R helps to remove LDL from the blood circulation. Additionally, the higher expression of LDL-R in the normal nondiabetic group and the lower LDL level in the G100 group suggest that GBR exerts its hypocholesterolemic effects through other mechanisms. This conclusion also applies to the BR, and G50 groups, which showed better hypocholesterolemic effects but similar effects on the expression of the LDL-R gene. APO A1 is the primary component of HDL cholesterol. The data suggest that the high HDL levels observed upon GBR consumption may be due in part to the induction of APO A1 gene expression. These data also suggest that mechanisms other than the transcriptional regulation of the APO A1 gene are likely involved in the mechanism by which GBR improves the HDL level because the expression levels of this gene in the control, BR and G50 groups were similar despite the improvement in the HDL levels of the latter 2 groups. 

The expression patterns in HEPG2 cells mirrored the expression findings in diabetic rats. The bioactive compounds used (ASG, GABA, oryzanol, and phenolics) clearly contributed to the upregulation of the LDL-R gene but did not result in expression levels similar to those under normal physiological conditions, confirming that other mechanisms are involved in the overall hypocholesterolemic effect of GBR. The expression patterns of the APO A1 gene also suggest that ASG, GABA, and phenolic extracts contributed to the upregulation of the expression of this gene by GBR, consistent with the findings of the animal study. The results of our animal study demonstrated that GBR can upregulate APO A1 gene expression to levels higher than those in normal nondiabetic rats, whereas the in vitro study revealed that ASG and phenolics were able to upregulate the expression of this gene more than insulin treatment did. Based on these results, it can be extrapolated that ASG and phenolics contributed to the ability of GBR to upregulate the expression of this gene to levels higher than those under normal physiological conditions. 

In our study, we demonstrate that the transcriptional regulation of the LDL-R and APO A1 genes may be involved in the hypocholesterolemic effects of GBR. It is apparent from our data and from the results of previous studies [17] that multiple mechanisms, both transcriptional and nontranscriptional, may be involved in these hypocholesterolemic effects. It should be noted, however, that most data on hypocholesterolemic effects of GBR are from animal studies, with very few short-term clinical studies. Although the findings from these clinical trials involving smaller number of subjects seem to mirror findings from animal studies [17], long-term clinical studies are still needed for a clinically relevant understanding of the hypocholesterolemic effects of GBR. In the meantime, however, GBR, with its multiple hypocholesterolemic mechanisms, may potentially serve as an important cholesterol-lowering food, especially in regions of the world where rice is the staple food [17].

## 5. Conclusions

There are many bioactive compounds in GBR, and synergism is apparently important in the hypocholesterolemic effects of GBR, which are mediated by transcriptional and nontranscriptional mechanisms. We have been able to demonstrate that the hypocholesterolemic effects of GBR in type 2 diabetic rats are partly mediated through the upregulation of the LDL-R and APO A1 genes. ASG, GABA, oryzanol, and phenolics from GBR contribute to the upregulation of the LDL-R gene, and ASG, GABA, and phenolics contribute to the upregulation of the APO A1 gene. Therefore, GBR could be an important lipid-lowering functional food, or its bioactive compounds could be developed into lipid lowering nutraceuticals that can be used to lower the risk of cardiovascular diseases.

## Figures and Tables

**Table:** (a)

Gene name [accession number]*	Primer sequence (with universal tag)	
	Forward	Reverse
LDLR [NM_175762]	AGGTGACACTATAGAATAATGAAGCCATTTTCAGTGCC	GTACGACTCACTATAGGGAAGGTGAACTTGGGTGAGTGG
HMGCR [NM_013134]	AGGTGACACTATAGAATATAGAGACGTCTGCGGTCCTT	GTACGACTCACTATAGGGATTAACCCATTGGAGGTGAGC
APO A1 [NM_012738]	AGGTGACACTATAGAATACAACTGGGACACTCTGGGTT	GTACGACTCACTATAGGGAATCTCCTTCGCGTTTTTGTG
Actb [NM_031144]^a^	AGGTGACACTATAGAATAGGCATCCTGACCCTGAAGTA	GTACGACTCACTATAGGGAAGACGCAGGATGGCATGAG
GAPDH [NM_017008]^a^	AGGTGACACTATAGAATACTGAGGACCAGGTTGTCTCC	GTACGACTCACTATAGGGAGAGGGCCTCTCTCTTGCTCT
PPIA [NM_017101]^a,∗^	AGGTGACACTATAGAATATTCTGTAGCTCAGGAGAGCA	GTACGACTCACTATAGGGATTGAAGGGGAATGAGGAAAA
KanR^b^		

^a^Housekeeping gene.  ^b^Internal control.  *Normalization gene.

**Table:** (b)

Gene name [Accession number]*	Primer sequence (with universal tag)	
	Forward	Reverse
LDLR [NM_000527]	AGGTGACACTATAGAATACAGGACGGCTACAGCTACCC	GTACGACTCACTATAGGGACTTATCCTTCACGAGGAAAGGA
HMGCR [NM_000859]	AGGTGACACTATAGAATAAATGGCAACAACAGAAGGTTGT	GTACGACTCACTATAGGGAGAAACGGATATAAAGGTTGCGT
APO A1 [NM_000039]	AGGTGACACTATAGAATATGTGTACGTGGATGTGCTCA	GTACGACTCACTATAGGGAGAGCTCCATCTCCTCCTGC
Actb [NM_001101]^a^	AGGTGACACTATAGAATAGATCATTGCTCCTCCTGAGC	GTACGACTCACTATAGGGAAAAGCCATGCCAATCTCATC
GAPDH [NM_002046]^a,∗^	AGGTGACACTATAGAATAAAGGTGAAGGTCGGAGTCAA	GTACGACTCACTATAGGGAGATCTCGCTCCTGGAAGATG
EEF1A1 [NM_001402]^a^	AGGTGACACTATAGAATACACACGGCTCACATTGCAT	GTACGACTCACTATAGGGACACGAACAGCAAAGCGA
KanR^b^		

^a^Housekeeping gene.   ^b^Internal control.   *Normalization gene.

**Table 2:** Concentration of acylated steryl glycoside (ASG), gamma amino butyric acid (GABA), oryzanol, and phenolics in germinated brown rice (GBR) in comparison to white rice (WR) and brown rice (BR).

Bioactive compound*	Rice type		
	GBR	BR	WR
ASG (mg/g)	0.465 ± 0.055	Undetected	Undetected
GABA (mg/g)	0.36 ± 0.04	0.09 ± 0.02	Undetected
Phenolics (GAE/g dw)	20.5 ± 0.01	3.17 ± 1.68	0.60 ± 0.45
Oryzanol (mg/100 g)	30.38–64.22	13.01–22.37	Undetected

GAE: gallic acid equivalent; dw: dry weight. *Concentration of all bioactive compounds in GBR was found to be significantly higher (*P* < 0.05) than WR or BR.

**Table 3:** Baseline parameters for the rat groups after the induction of diabetes and the level of food consumption of each group.

Rat groups	Glucose (mmol/L)*	Total cholesterol (mmol/L)*	HDL cholesterol (mmol/L)*	Triglycerides (mmol/L)*	LDL cholesterol (mmol/L)*	Calories (Kcal/100 g pellet)	Food consumption (kcal/100 g body weight/day)*
Normal nondiabetic	4.6 ± 0.5^a^	1.88 ± 0.1^a^	0.48 ± 0.09^a^	0.60 ± 0.07^a^	0.30 ± 0.03^a^	335	30.5 ± 3.7^a^
Control	14.9 ± 2.2^b^	2.50 ± 0.06^b^	0.47 ± 0.07^a^	1.00 ± 0.31^b^	0.38 ± 0.05^a^	548	34.0 ± 6.0^a^
WR	19.1 ± 2^b^	2.50 ± 0.06^b^	0.48 ± 0.04^a^	1.09 ± 0.28^b^	0.39 ± 0.11^a^	554	33.2 ± 8.3^a^
BR	18.4 ± 2.8^b^	2.34 ± 0.30^b^	0.31 ± 0.11^a^	1.02 ± 0.11^b^	0.32 ± 0.11^a^	554	30.5 ± 6.7^a^
G50	17.3 ± 2.5^b^	2.21 ± 0.07^b^	0.45 ± 0.09^a^	1.32 ± 0.35^b^	0.46 ± 0.04^b^	554	33.2 ± 8.3^a^
G100	17.3 ± 1.8^b^	2.32 ± 0.22^b^	0.44 ± 0.05^a^	1.23 ± 0.16^b^	0.44 ± 0.05^b^	560	35.3 ± 7.3^a^

*Values represent the mean ± SD. Values with the same letter in any given column are not significantly different (*P* > 0.05). ASG: acylated steryl glycoside; GBR: germinated brown rice; WR: white rice.

## Abbreviations

ASG: Acylated steryl glycoside
APO A1: Apolipoprotein A1
BR: Brown rice
GABA: Gamma amino butyric acid
GBR: Germinated brown rice
HDL: High-density lipoprotein
LDL: Low-density lipoprotein
LDL-R: Low-density lipoprotein receptor
WR: White rice.
